# Modulation of Somatosensory Evoked Potentials During Music and Pink Noise Exposure

**DOI:** 10.3390/brainsci16080802

**Published:** 2026-07-30

**Authors:** Ana Maria Cebolla, Pablo Ruiz, Martin Colot, Cédric Simar, Viktoriya Vitkova, Olivier Van Hove, Saïd-Iraj Hashemi, Guy Cheron

**Affiliations:** 1 Movement Neurophysiology Laboratory (NeuroMove), ULB Neuroscience Institute, Université Libre de Bruxelles, 1050 Bruxelles, Belgium; pablo.ruiz@ulb.be (P.R.); viktoriya.vitkova@ulb.be (V.V.); olivier.van.hove@ulb.be (O.V.H.); said-iraj.hashemi@ulb.be (S.-I.H.); guy.cheron@ulb.be (G.C.); 2 Machine Learning Group, Université Libre de Bruxelles, 1050 Bruxelles, Belgium; martin.colot@ulb.be (M.C.); cedric.simar@ulb.be (C.S.)

**Keywords:** somatosensory evoked potential, EEG, music, pink noise, multimodal, N30, P45

## Abstract

**Highlights:**

**What are the main findings?**

- Middle-latency somatosensory evoked potentials were modulated by auditory stimulation.
- Music and pink noise produced distinct temporal patterns of middle-latency SEP modulation.

**What are the implications of the main findings?**

- Early lemniscal somatosensory processing remained unchanged during auditory stimulation.
- Auditory context shapes middle-latency somatosensory processing in a stimulus-dependent manner.

**Abstract:**

Background: Music and pink noise share spectral similarities; however, their cognitive and emotional properties differ. Previous studies using magnetoencephalography have shown that somatosensory evoked components occurring before 60 ms are not modulated by concomitant musical auditory stimulation. To date, no study has examined the effects of pink noise on somatosensory evoked potentials. Objective: This study investigated, using electroencephalography (EEG), whether continuous auditory stimulation, either music or pink noise, can modulate early (before 60 ms) somatosensory evoked potentials elicited by electrical stimulation of the median nerve. Methods: Somatosensory evoked potentials elicited by right median nerve electrical stimulation were recorded through electroencephalogram in two groups of participants (MUSIC and NOISE) in four conditions (pre, during 1, during 2, and after auditory stimulation). In addition, a control group underwent three consecutive SEP recordings, temporally aligned with the intervention groups’ sequence. A permutation-based ANOVA (10,000 permutations) was performed over the entire 10–55 ms time window for the F1, C1, and CP3 channels, followed by corrected post hoc comparisons (pre vs. During1, pre vs. During2, pre vs. post). Results: A significant increase in the amplitude of the P45 component was observed in the left central site during noise exposure, with post hoc comparisons indicating increased amplitudes in during1 and during2 relative to pre, with a larger effect size in during2. Music resulted in a reduction in the early part (~30 ms) of the left centroparietal positive brain response, as highlighted by post hoc comparisons between during2 and preconditions. In the control group, no comparisons were significant, indicating the absence of reliable differences between repetitive recordings. Conclusion: Middle-latency SEP components (30–45 ms) showed distinct amplitude modulations during both music and pink noise auditory exposure.

## 1. Introduction

Cognitive flexibility relies on the capacity to discriminate, among numerous sensory stimuli originating from multiple origins, the information most relevant for selecting the execution of the most appropriate behavior [1,2]. This ability to select among different stimuli is initially facilitated by the existence of anatomically distinct receptors and pathways according to the different sensory modalities. However, once their integration becomes structurally possible, through synaptic convergence or other physiological mechanisms, the problem of prioritizing information arises.

Studies conducted in the field of sensory gating [3,4,5], in which somatosensory evoked potentials (SEPs) elicited by electrical stimulation of the median nerve are examined in conjunction with the execution of a voluntary movement, concomitant cutaneous stimulation, or motor imagery, have made it possible to differentiate SEP components that are insensitive to these various interferences from those that are modified (enhanced, reduced, or suppressed) [6,7,8,9,10].

The divergence points observed on SEP superimposition waveforms separating traces obtained under control conditions from those recorded in gating conditions provide information about the timing of the underlying processes and, consequently, about the neuronal structures involved or not in the physiological interactions. The various SEP components elicited by median nerve stimulation offer the advantage of having been extensively studied both in different gating paradigms and in clinical neurology [11,12,13,14,15,16,17,18].

Auditory stimulation devices are commonly used to promote relaxation and sleep, although their effectiveness remains insufficiently established and understood [19]. In humans, evidence regarding the modulation of early (before 60 ms) somatosensory evoked potentials (SEPs) by auditory stimulation is scarce. Previous studies in MEG have reported that middle-latency somatosensory evoked fields components, occurring between approximately 35 and 60 ms, were not altered by music exposure [20,21]. However, as MEG is predominantly sensitive to tangential sources, activity from radially oriented generators may not have been fully captured.

While both music and pink noise can display 1/f-like spectral similarities, they fundamentally diverge in terms of temporal structure, hierarchical organization [22,23,24,25], and cognitive and emotional content [26,27]. Moreover, EEG activity exhibits pink-noise-like properties [28], and steady pink noise exposure has been shown to reduce brain wave complexity and to promote more stable sleep, thereby improving sleep quality [29].

In the present study, we investigated whether continuous auditory stimulation, music or pink noise, could modulate early somatosensory evoked potentials elicited by electrical stimulation of the median nerve.

### Ethics Declaration

The study was approved by the Ethics Committee of the Brugmann University Hospital, Brussels (Ref. CE2023/182), and conformed to the Declaration of Helsinki. All participants signed informed consent forms. All data were collected, processed, and analyzed in compliance with the General Data Protection Regulation (GDPR; Regulation (EU) 2016/679).

## 2. Participants, Materials and Methods

Forty-five healthy subjects (23 female/22 male, 22.5 ± 1.5 years old) participated in this study. All participants were right-handed and had no history of neurological or psychiatric disorders. They were progressively assigned to the groups of fifteen (“Music”, “Noise”, and “Control”), with balanced sex distribution across groups, with the control group included in a second phase.

Recordings were conducted in a quiet, temperature-controlled room where participants were comfortably seated in an armchair, with their arms and forearms resting on pillows on the armrests in a relaxed and comfortable position. Somatosensory stimulation consisted of 1000 square electrical pulses of 2 ms duration, with a frequency of 2.15 Hz, delivered through a pair of Ag-AgCl electrodes cup to the right median nerve at the wrist. The intensity was individually adjusted to elicit small visible thumb twitches without discomfort. Such somatosensory stimulation was delivered during four conditions by a Digitimer Constant Current Stimulator DS7A (Digitimer Ltd., Hertfordshire, UK). The “PRE” condition was recorded at rest before sound administration (music or noise). After a five-minute break, the “During1” and “During2” conditions were recorded within the 30-min sound exposure (“During1” starting at 3 min after sound onset and “During2” starting at 17 min). The “POST” condition was recorded after a five-minute break following the end of sound administration.

For the Music group, every participant pre-selected a list of preferred songs from a commercial music streaming platform prior to the experiment. For the Noise group, participants were administered pink noise from the same commercial streaming platform. Pink noise was selected rather than white noise because it has a 1/f spectral profile that more closely resembles that of music and many natural acoustic environments. Both types of sounds were delivered using the same smartphone, which was placed on a table in front of the participant at around 70 cm with the same volume setting across the recordings.

Participants in the control group underwent three consecutive recordings, temporally aligned with the sound sequence of the Music and Noise groups, with the 1st, 2nd, and 3rd recordings corresponding to the pre, during1, and during2 conditions, respectively, and without auditory stimulation. The control group was specifically designed to evaluate the stability of SEP activity across repeated recordings performed without auditory stimulation, thereby allowing assessment of potential temporal drift or habituation effects independently of sound exposure.

All participants were instructed not to pay attention to the electrical stimulation, to relax, and to keep their eyes closed except during breaks. The same instructions were given to all the participants.

EEG signals were recorded during the four conditions using the ANT Neuro mylab™ (ANT Neuro BV, Hengelo, The Netherlands), (64 Ag/AgCl sintered ring electrodes and shielded co-axial cables, 10–20 electrode placement, CPz referenced) at 4096 Hz. Scalp electrode impedances were measured and kept below 5 KΩ. The EOG electrode provided by the EEGcap was placed on the lobe of the left ear for later offline re-referencing. The ANT amplifier received the temporal triggers of the electrical stimuli delivery from the DS7A device, meaning that the related events were directly recorded with the EEG signals.

### Somatosensory Evoked Potentials (SEP): Data Treatment, Analyses and Statistics

Raw EEG data were imported to EEGLAB software (version 2025.1.0) (Swartz Center for Computational Neuroscience, University of California San Diego, La Jolla, CA, USA) for offline data treatment and subsequent statistics [30]. Preprocessing procedures were applied consistently across participants and experimental conditions following the same EEGLAB-based analysis pipeline. Signals were kept at a 4096 Hz sampling rate, corresponding to a temporal resolution of approximately 4.1 samples per millisecond. Visual inspection for rejection of artefactual portions of EEG was performed after a 0.1 Hz high pass filter was applied, and a notch filter when necessary. EEG signals were re-referenced to the left earlobe electrode. Contralateral to the electrical stimulation, SEP traces were calculated at F1, C1, and CP3 electrodes to assess left frontal, left central, and left centroparietal SEP activity, respectively. These electrodes were selected a priori based on the well-established contralateral topography of median nerve SEP responses and therefore constituted predefined physiologically relevant regions of interest rather than exploratory whole-scalp comparisons. Accordingly, no additional correction for multiple comparisons across electrodes was applied, as these analyses were hypothesis-driven and restricted to predefined regions of interest. SEP traces were obtained by averaging (950 artifact-free epochs minimum per participant per condition) of baseline-corrected epochs extracted from −0.05 s to 0.1 s with respect to the events of the electrical stimulation.

The latencies and amplitudes of the P14 far-field potential and of the N20, N30, and P45 components were verified for each condition: P14 and N30 in the F1 channel, N20 in the CP3 channel, and P45 in the C1 channel. For descriptive analysis, the peaks (maximum for P or minimum for N) and their peak latencies were assessed in the 13.5 ms to 14.5 ms period for P14, in the 18 ms to 21 ms period for N20, in the 28 ms to 32 ms period for N30, and in the 43 ms to 47 ms period for the P45 component. Next, peak-to-peak amplitudes were assessed for the N30 component (P22 to N30) in the F1 channel and for the P45 component (N30 to P45) in the C1 channel. As the P14 far field is the first deflection in such cephalic reference montage, its peak-to-peak amplitude was calculated with respect to its onset, corresponding to the minimum peak value in the preceding 9.5 ms to 10.5 ms period. In the same way for the N20 component, a feature marking the first arrival of the sensory volley to the neocortex, its peak-to-peak amplitude was calculated with respect to the maximum peak value in the preceding 17 ms to 18 ms period.

For inferential analyses, permutation-based ANOVAs (10,000 permutations) were performed separately for each channel across the 10–55 ms time interval, providing a continuous sample-by-sample temporal assessment of condition effects. This approach allowed robust statistical inference without relying on theoretical parametric distributions for significance testing. Test statistics (F or t values) were computed using standard parametric formulations, while statistical significance was assessed across time using permutation-based procedures. Holm correction was applied across all analyzed time points to control the family-wise error rate associated with multiple comparisons over time. Reported significant results therefore are obtained from the continuous permutation-based analysis and do not directly correspond to isolated latency-specific tests. Due to the numerical resolution afforded by the 10,000 permutations, the minimum attainable *p*-value was approximately 0.0001.

Post hoc comparisons between conditions were then conducted within a common narrower time window of interest (27 ms to 49 ms), encompassing the temporal range where condition-related modulation effects were observed in the initial permutation-based ANOVA. Holm correction was applied across time points to control for multiple comparisons over time, and Bonferroni correction was used for post hoc contrasts. Statistical significance was set at *p* < 0.05, with Bonferroni correction applied for post hoc comparisons (adjusted threshold *p* < 0.0167). For significant post hoc intervals, effect sizes are provided using Cohen’s dz for paired comparisons.

## 3. Results

**Figure 1:**
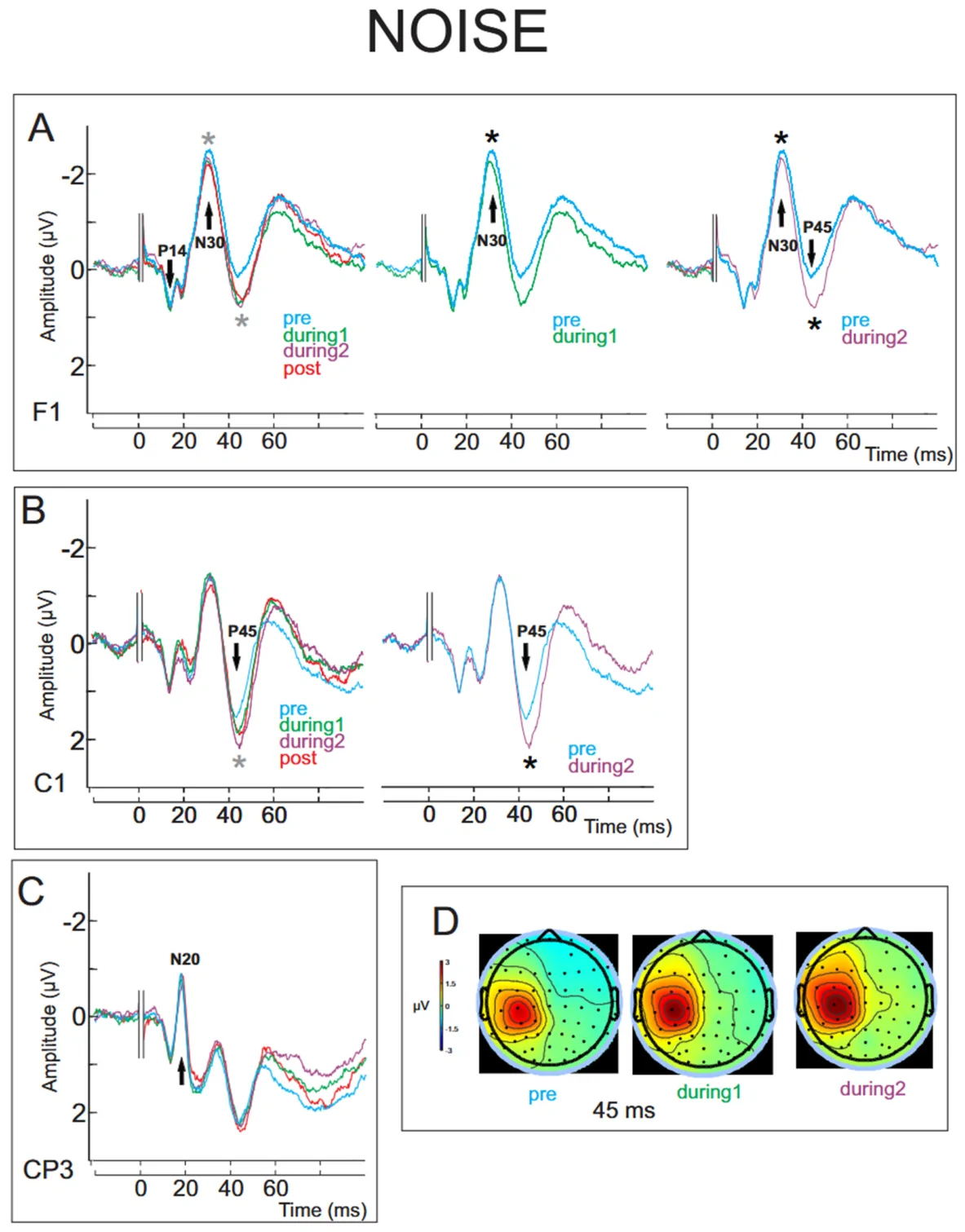
(**A**) Grand-averaged SEP waveforms at electrode F1 in the Noise group across conditions (pre: blue; during1: green; during2: violet; post: red). Light grey asterisks indicate the approximate locations of significant main effects of condition identified by the permutation-based ANOVA. Black asterisks indicate the approximate locations of significant Bonferroni-corrected post hoc differences. The exact significant time intervals are reported in the Results (Section 3). (**B**) Grand-averaged SEP waveforms at electrode C1 across the same conditions. (**C**) Grand-averaged SEP waveforms at electrode CP3 across the same conditions. (**D**) For descriptive purposes, scalp topography illustrating the larger contralateral central positivity observed at 45 ms from the pre to the during2 conditions.

**Figure 2:**
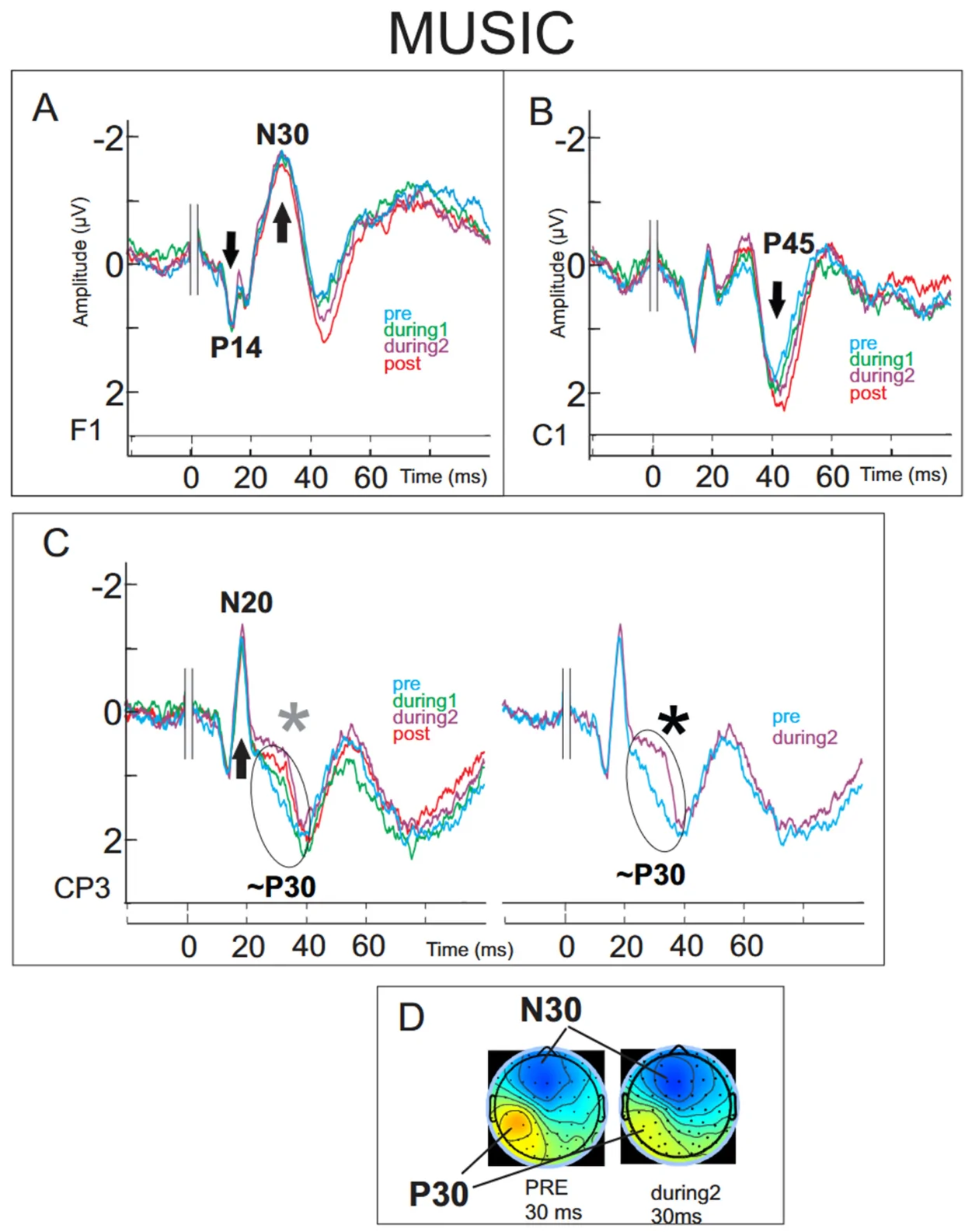
(**A**) Grand-averaged SEP waveforms at electrode F1 in the Music group across conditions (pre: blue; during1: green; during2: violet; post: red). (**B**) Grand-averaged SEP waveforms at electrode C1 across the same conditions. (**C**) Grand-averaged SEP waveforms at electrode CP3 across the same conditions. Light grey asterisks indicate the approximate locations of significant main effects of condition identified by the permutation-based ANOVA. Black asterisks indicate the approximate locations of significant Bonferroni-corrected post hoc differences. The exact significant time intervals are reported in the Results (Section 3). (**D**) For descriptive purposes, scalp topography illustrating the decrease in contralateral centroparietal positivity at 30 ms (P30) from the pre to during2 conditions, together with the concomitant frontal negativity within the N30 time range.

**Figure 3:**
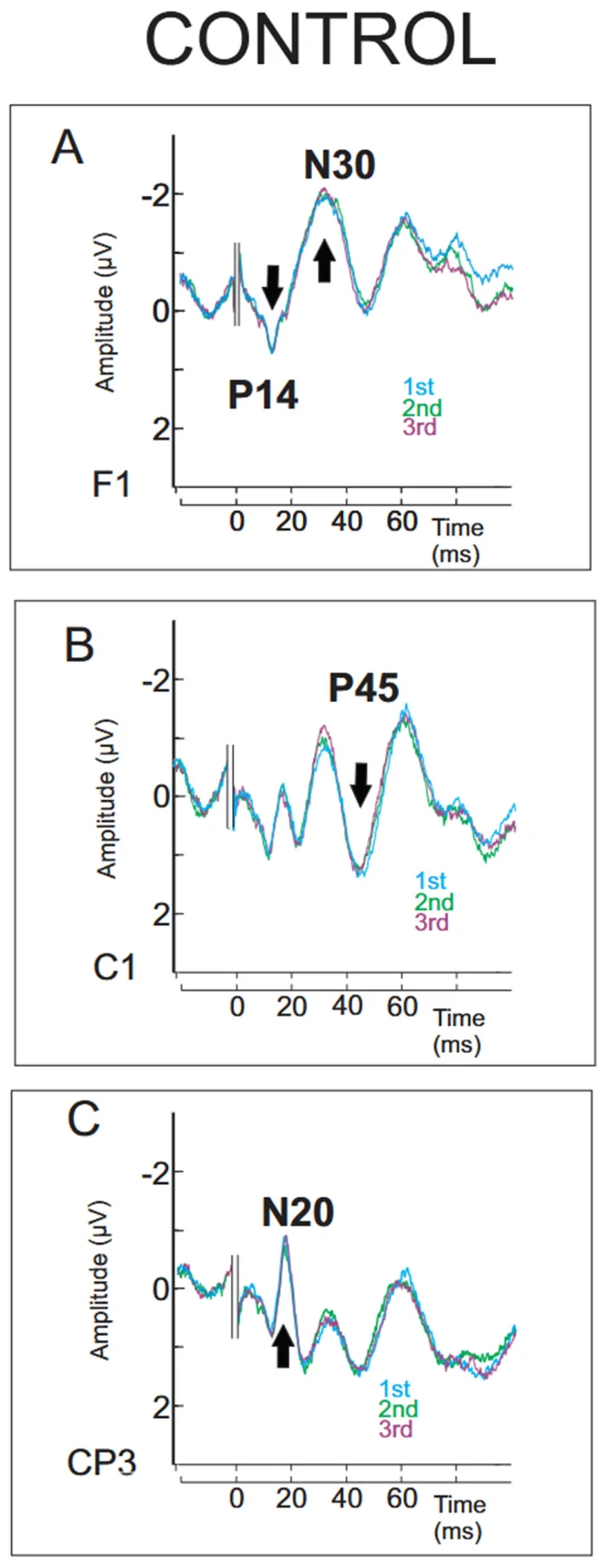
(**A**) Grand-averaged SEP waveforms at electrode F1 in the Control group for the 1st, 2nd, and 3rd recordings (blue, green, and violet, respectively). (**B**) Grand-averaged SEP waveforms at electrode C1 for the same recordings. (**C**) Grand-averaged SEP waveforms at electrode CP3 for the same recordings.

Descriptive values of amplitudes and latencies for the P14 far-field potential and classical components N20, N30, and P45 are reported in the Supplementary Section (Tables S1–S3) for each condition and group. Grand-averaged SEP traces superimposed across the four conditions (PRE, during1, during2, POST) are illustrated for the Noise group in Figure 1, and for the Music group in Figure 2. Significant effects identified by the initial permutation-based ANOVA are indicated by grey asterisks, whereas significant post hoc differences are marked by black asterisks. Grand-averaged SEP traces from the 1st, 2nd, and 3rd recordings for the control group are superimposed in Figure 3. Supplementary Figures S1–S6 illustrate the continuous temporal evolution of permutation-based ANOVA *p*-values across the analyzed SEP time windows. Supplementary Figures S7–S10 provide the corresponding permutation-based post hoc *p*-value distributions associated with the reported significant effects.

In the Noise group, SEP traces at the F1 electrode (Figure 1A and Figure S1) showed a significant effect of condition from 36.87 to 38.33 ms (permutation-based *p* = 0.02; peak F (3,42) = 7.12 at 37.87 ms) and from 44.19 to 47.85 ms (permutation-based *p* < 0.02; peak F (3,42) = 10.38 at 45.65 ms). Post hoc comparisons (Figure S7) (Bonferroni-corrected threshold *p* < 0.0167) revealed a significant difference between pre and during1 around 37–38 ms (peak at 37.84 ms, t (14) = −3.56, dz = −0.61). Significant post hoc differences between pre and during2 were observed mainly in two intervals (Figure S8): an early effect around 36–39 ms (peak at 36.87 ms, t (14) = −5.45, dz = −1.05) and a later effect around 43–47 ms (peak at 45.65 ms, t (14) = −5.62, dz = −1.33).

At the C1 electrode (Figure 1B and Figure S2), SEP traces showed a significant effect of condition within a short time interval from 45.65 to 46.39 ms (permutation-based *p* = 0.02; peak F (3,42) = 7.24 at 46.14 ms). Post hoc comparisons (Figure S9) revealed a significant difference between pre and during2 over the 42.72 to 46.88 ms interval (peak effect at 45.65 ms, t(14) = −5.62, dz = −1.06). For descriptive purposes, a topographical map of the grand-averaged potential distribution at 45 ms is presented in Figure 1D, illustrating a larger contralateral central positivity (in red) extending from the pre to the during2 condition. SEP traces at the CP3 (Figure 1C) electrode revealed no significant differences.

In the Music group, SEP traces at the CP3 electrode (Figure 2C and Figure S3) showed a significant effect of condition from 31.25 to 34.18 ms (permutation-based *p* < 0.02; peak F(3,42) = 9.21 at 31.74 ms). Post hoc comparisons (Figure S10) revealed a significant difference between pre and during2 over the 27.59 to 34.91 ms interval (peak at 31.74 ms, t(14) = 6.39, dz = 1.31). This significant effect corresponded to a reduction in the positive voltage at the contralateral centroparietal site (mean amplitudes: 1.55 ± 1.28 µV for pre and 0.78 ± 1.40 µV for during2), within the 31–34 ms latency range. No other significant differences were observed at any other electrode or for any other contrast. For descriptive purposes, a topographical map of the grand-averaged potential distribution at 30 ms is presented in Figure 2D, illustrating a contralateral centroparietal positivity (in red) decreasing from the pre to the during2 condition. The concomitant frontal negativity within the N30 time range is illustrated in blue.

In the Control group, no significant effect of recording order was observed across the 1st, 2nd, and 3rd recordings (Figure 3 and Figures S4–S6), which were temporally aligned with the sequence used in the Noise and Music groups, indicating stable SEP responses over time (permutation-based *p* = 0.99 F(2,24) = 0.01 at 39.31 ms in F1; *p* = 0.99 F(2,24) = 0.01 at 45.90 ms in C1; *p* = 0.99 F(2,24) = 0.01 at 37.35 ms in CP3).

## 4. Discussion

The present study examined the effects of different sound exposures on somatosensory evoked potentials, revealing distinct patterns of modulation across groups. While SEP responses remained stable over time in the control group, noise exposure was associated with temporally extended modulations, particularly within the 30–50 ms latency range. In contrast, music exposure induced a more focal and temporally selective effect, primarily observed at the CP3 electrode within an earlier latency window (~30 ms), driven by differences between the pre and during2 conditions.

In the control group, SEP traces further confirmed the overall stability of early somatosensory processing across repeated recordings. No significant effect of condition on the P14 or N20 components was observed in any of the groups. The stability of the P14 component indicates that early lemniscal somatosensory processing remains unaffected [14] by concurrent auditory stimulation. Cross-modal modulations are generally ascribed to non-lemniscal thalamocortical and to corticocortical connections across sensory and associative cortices [31,32,33]. It has been demonstrated that somatosensory (transcutaneous electrical) stimulation of the rat hind paw paired with auditory stimulation (noise bursts) elicited unit cell discharges in non-lemniscal auditory nuclei of the thalamus, while also inducing subthreshold activity modulations in lemniscal auditory thalamic nuclei [34]. In the present study, the N20 obligatory component, which is the earliest response in the postcentral receiving primary somatosensory cortex [3,12,35,36], also remained unchanged across the four conditions. This indicates that the present observed SEP amplitude changes are neither of peripheral origin nor centripetal gating, which is produced by competition of peripheral information from joint, cutaneous, and muscle spindles to the central process [9,10,37,38]. It also indicates that the underlying mechanisms of the effects observed in this study are unlikely to relate to those of the hypnosis-like states or hypnotic suggestion, in which modulations of N20 amplitude have previously been reported and interpreted as a signal intensity reduction at the level of the thalamocortical radiations during hypnosis [39].

In the noise group, SEP modulations emerged specifically during the auditory exposure, primarily at the F1 electrode and, to a lesser extent, at C1. At F1, significant differences between the pre and both during conditions were observed within two temporally distinct intervals (~37–38 ms and ~44–47 ms), suggesting a modulation of early frontal SEP components, sparing the N30 peak. Notably, the strongest effect was observed during the 2nd sound recording in the later time window (~45 ms) in the during2 condition, indicating that the influence of noise exposure may not only persist but also intensify over time, particularly at the level of recurrent and integrating processing stages where ongoing brain dynamics and conscious perception may have contributed to this effect [40]. At the C1 electrode, a more temporally restricted modulation was observed around the latency of the P45 component, again driven by differences between pre and during2. This effect, occurring at a latency comparable to that identified at F1, further supports the involvement of mid-latency somatosensory processing under noise exposure. In contrast, the absence of any significant effect at CP3 indicates that these modulations do not extend to the earliest cortical stage indexed by the N20 component, but rather involve later processing stages, potentially reflecting top-down influences such as attentional allocation or changes in cortical excitability induced by the auditory context [41]. These mechanisms remain speculative and should be interpreted with caution, given the lack of behavioral or psychological measures.

In contrast to the noise group, the music group exhibited a more temporally focal modulation, restricted to the CP3 electrode and occurring within the 31–34 ms latency range. Because this effect was observed as a positivity at CP3 rather than as the frontal negative deflection classically associated with the N30 component, it is more cautious to interpret it as a modulation occurring within the N30 time range but overlapping with positive deflections described in the literature as P30 and considered distinct from the classical N30 [7,42,43]. The fact that no concomitant effect was observed at F1 further supports this interpretation. Nevertheless, the large effect size associated with the pre–during2 contrast suggests that music exposure induced this robust and temporally specific change in somatosensory processing beyond the earliest afferent stages, while differing from the later pattern observed under noise exposure.

Interestingly, our results differ from a previous study [20] investigating the effects of music and the effects of continuous visual stimulation on somatosensory evoked magnetic fields elicited by homogeneous electrical stimuli on the median nerve; the study showed that the contralateral 35–60 ms in latency SEFs components were significantly enhanced by the visualization of an animation video, but they were not modified by the music exposure. Additionally, in a later study taking into account selective attention, the same group showed that contralateral 33–75 ms in latency SEFs components were enhanced by both attention alone and, visual alone stimulation, and that the SEFs were even more enhanced by the combination of both attention and visual stimulation; but, as in their first study, music did not produce any significant modification in such SEFs [21]. The explanation for such lack of change proposed was that, as in the parietal lobe, polymodal neurons responding to auditory and somatosensory stimulation are much fewer in number than those responding to visual and somatosensory stimulation [44,45,46]; therefore, their activation might not be large enough to induce a significant change in the SEFs. This explanation may be consistent with the present findings, as the observed modifications were primarily driven by differences between the pre and during2 conditions, suggesting that longer or repeated exposure to music may progressively engage a larger neural population. In addition, a methodological consideration should be considered because MEG is predominantly sensitive to tangential sources; it is possible that the observed effects partly arise from radially oriented generators that were not fully captured.

Music and pink noise may share 1/f-like spectral properties; however, they fundamentally differ in their temporal structure, hierarchical organization [22,23,24,25], and cognitive and emotional content [26,27,29]. Consequently, the differential SEP modulations observed in the present study cannot be attributed solely to differences in spectral content or multisensory interactions, and may also reflect differences in emotional valence, familiarity, temporal predictability, or attentional engagement. Psychological data have previously demonstrated that music induces significant relaxation responses, regardless of whether it is self-selected by the participant or chosen by the experimenter [47], and that classical and self-selected relaxing music leads to higher relaxation levels than hard rock music [48], but that only preferred music produced a reduction in anxiety levels approached by systolic and diastolic blood pressure measurements [49]. Furthermore, previous studies have shown that brain reactivity, as assessed by EEG power modulations, is consistent across individuals when music is self-selected, with comparable subjectively evoked emotions, despite interindividual variability in EEG frequency bands related to individual EEG characteristics [50]. fMRI studies have proposed that among the cortical substrates of the subjective feeling evoked by music, the secondary somatosensory cortex has a major role in encoding emotion precepts as sustained by its anatomical connections with limbic–paralimbic structures, sensory-interoceptive and visceromotor structures (insula, anterior cingulate cortex), and motor cortical and subcortical structures [51,52]. As the modulation observed in the music group was located at CP3 around ~30 ms, and not at F1 nor within the typical N30 pattern, this suggests a specific and temporally selective change in somatosensory processing, mainly driven by differences between the pre and during2 conditions. One possible interpretation is that music induces a modulation beyond the earliest afferent stages but not corresponding to frontal generators (previously proposed for the N30 generators [43]), maybe reflecting a local parieto-central cortical reconfiguration.

The works of Desmedt and Tomberg [53,54,55] described in detail the enhancement of the SEP P40 amplitude as a cognition-related potential during selective attention to randomly intermixed electrical stimulations delivered to the finger in an oddball task, in which a cognitive P30 component emerged during more demanding stimulus discrimination. P30 and P40 were proposed to prime somatic areas through activity enhancement in parietal areas 1, 2, and 5, facilitating subsequent conscious perception of the target input and preceding the classical P300 post-decision closure [56,57]. However, neither the present protocol nor the original study by Lam et al. (1999 [20] used an oddball paradigm, but rather a homogeneous series of electrical stimuli. In this context, it could be speculated that the P45 modifications observed in the group exposed to noise might correspond to the cognitive P40 elicited by homogeneous electrical stimuli that participants may have neglected while daydreaming or reading [58], possibly reflecting early attentional effects induced by noise exposure that may contribute to the attenuation of the concomitant somatosensory electrical stimuli, although alternative explanations related to differences in auditory context cannot be excluded.

Multisensory integration at the earliest stages of cortical processing is supported by anatomical evidence of direct, monosynaptic connections between primary sensory cortices [59,60]. In addition, there is growing evidence that primary sensory cortices contain distinguishable spatial patterns of activity for each sensory modality [61]. For example, somatosensory stimulation has been shown to modulate neural activity in the primary auditory cortex in primates, including humans [62]. As discussed earlier, the fact that the N20 remained stable across conditions discards the primary somatosensory cortex as a source of the observed changes. This is in line with recent electrophysiological recordings in mice using in vivo two-photon calcium imaging, which have demonstrated that sound-evoked spiking activity is present in only an extremely small fraction of cells in the primary somatosensory cortex, and that such activity does not encode auditory stimulus identity and, even more, that the primary somatosensory cortex does not code audio-tactile cross-sensory input’s features [63].

It is also possible that the modulations observed in our study involve additional cortical regions different than the classical sensorimotor ones at such early latencies. Source modelling analysis has supported that the two different mechanisms proposed to generate the N30 component power increase and phase locking across EEG trials [64] are spatially localized in overlapping areas in the precentral cortex, namely the motor cortex and the premotor cortex. From this common region, the generator of the N30 event-related potential also involved the prefrontal cortex [43].

Several limitations should be acknowledged. First, the absence of complementary psychophysical assessments does not fully allow us to disentangle potential differences in emotional states induced by the auditory conditions. Consequently, the relative contribution of emotional, attentional, familiarity-related, and low-level acoustic factors to the observed SEP modulations cannot be determined from the present data. While attentional modulation cannot be entirely ruled out, the use of monotonous electrical stimulation rather than an oddball paradigm limits the extent to which attention can be inferred from the present data. In addition, the relatively long and repetitive nature of the task may have led to some degree of fatigue or reduced engagement over time, although this was similar across groups. In addition, no formal a priori power analysis was performed; therefore, non-significant findings should be interpreted cautiously, as smaller effects may require larger cohorts to be reliably detected. Moreover, the auditory stimuli were not characterized in terms of emotional valence, arousal, or familiarity, which limits the interpretation of the specificity of the observed effects. In addition, the auditory stimuli were not characterized in detail in terms of their acoustic properties, and no objective sound pressure level calibration was performed. Consequently, perceived loudness may have varied across participants, making it difficult to determine whether the observed effects were specifically related to the nature of the stimuli or partly driven by differences in their low-level physical characteristics and subjective auditory perception. Because participants listened to self-selected music, acoustic features such as tempo, rhythmic complexity, spectral content, and dynamic range were not standardized across individuals, which may also have contributed to inter-individual variability. Consequently, the relative contribution of low-level acoustic properties and higher-order cognitive or emotional factors cannot be fully disentangled in the present study. Furthermore, although the control group showed consistent stability across repeated recordings, the absence of a formal a priori power analysis warrants cautious interpretation of non-significant findings, which should not be considered as evidence for the absence of smaller effects. Finally, the present analyses were conducted at the sensor level; future studies could incorporate high-density electrode analyses and source-level approaches to better characterize the underlying neural generators.

## 5. Conclusions

Altogether, these results suggest that auditory context does not uniformly affect middle somatosensory processing but rather shapes its temporal dynamics in a condition-dependent manner, with noise and music exerting qualitatively different influences along the processing stream.

## Data Availability

The data presented in this study are available on request from the corresponding author because the data are part of an ongoing study.

## Supplementary Materials

The following supporting information can be downloaded at: https://www.mdpi.com/article/10.3390/brainsci16080802/s1, Figure S1: Permutation-based ANOVA *p*-values across time for the SEP analysis at electrode F1 in the Noise group. Both panels display the same analyzed time window, but with complementary *p*-value ranges. The upper panel focuses on *p*-values above 0.08, whereas the lower panel provides a magnified view of the lower *p*-value range (0–0.08). The horizontal black line indicates the significance threshold (*p* = 0.05). Grey bars at the bottom indicate time intervals showing a significant main effect of condition after Holm correction for multiple comparisons across time; (*p*-values displayed as 0 correspond to values below the numerical resolution afforded by the 10,000 permutations and should therefore be interpreted as *p* < 0.0001); Figure S2: Permutation-based ANOVA *p*-values across time for the SEP analysis at electrode C1 in the Noise group. Both panels display the same analyzed time window, but with complementary *p*-value ranges. The upper panel focuses on *p*-values above 0.08, whereas the lower panel provides a magnified view of the lower *p*-value range (0–0.08). The horizontal black line indicates the significance threshold (*p* = 0.05). Grey bars at the bottom indicate time intervals showing a significant main effect of condition after Holm correction for multiple comparisons across time; (*p*-values displayed as 0 correspond to values below the numerical resolution afforded by the 10,000 permutations and should therefore be interpreted as *p* < 0.0001).; Figure S3: Permutation-based ANOVA *p*-values across time for the SEP analysis at electrode CP3 in the Music group. Both panels display the same analyzed time window, but with complementary *p*-value ranges. The upper panel focuses on *p*-values above 0.08, whereas the lower panel provides a magnified view of the lower *p*-value range (0–0.08). The horizontal black line indicates the significance threshold (*p* = 0.05). Grey bars at the bottom indicate time intervals showing a significant main effect of condition after Holm correction for multiple comparisons across time; (*p*-values displayed as 0 correspond to values below the numerical resolution afforded by the 10,000 permutations and should therefore be interpreted as *p* < 0.0001); Figures S4–S6: Permutation-based ANOVA *p*-values across time for the SEP analysis at electrodes F1, C1, and CP3, respectively, in the Control group. Both panels display the same analyzed time window but with complementary *p*-value ranges. The upper panel focuses on *p*-values above 0.08, whereas the lower panel provides a magnified view of the lower *p*-value range (0–0.08). The horizontal black line indicates the significance threshold (*p* = 0.05). No time intervals survived Holm correction for multiple comparisons across time. *p*-values displayed as 0 correspond to values below the numerical resolution afforded by the 10,000 permutations and should therefore be interpreted as *p* < 0.0001; Figure S7: Permutation-based post hoc *p*-values across time for the comparison between the Pre and During1 conditions at electrode F1 in the Noise group. Both panels display the same analyzed time window but with complementary *p*-value ranges. The upper panel focuses on *p*-values above 0.08, whereas the lower panel provides a magnified view of the lower *p*-value range from 0 to 0.08. The horizontal black line indicates the nominal significance threshold (*p* = 0.05), whereas the horizontal grey line indicates the Bonferroni-corrected threshold applied to planned post hoc comparisons (*p* = 0.0166). Grey bars at the bottom indicate time intervals remaining significant after Holm correction across time points within the analyzed post hoc time window. *p*-values displayed as 0 correspond to values below the numerical resolution afforded by the 10,000 permutations and should therefore be interpreted as *p* < 0.0001; Figure S8: Permutation-based post hoc *p*-values across time for the comparison between the Pre and During2 conditions at electrode F1 in the Noise group. Both panels display the same analyzed time window but with complementary *p*-value ranges. The upper panel focuses on *p*-values above 0.08, whereas the lower panel provides a magnified view of the lower *p*-value range from 0 to 0.08. The horizontal black line indicates the nominal significance threshold (*p* = 0.05), whereas the horizontal grey line indicates the Bonferroni-corrected threshold applied to post hoc comparisons (*p* = 0.0166). Grey bars at the bottom indicate time intervals remaining significant after Holm correction across time points within the analyzed post hoc time window. *p*-values displayed as 0 correspond to values below the numerical resolution afforded by the 10,000 permutations and should therefore be interpreted as *p* < 0.0001; Figure S9: Permutation-based post hoc *p*-values across time for the comparison between the Pre and During2 conditions at electrode C1 in the Noise group. Both panels display the same analyzed time window but with complementary *p*-value ranges. The upper panel focuses on *p*-values above 0.08, whereas the lower panel provides a magnified view of the lower *p*-value range from 0 to 0.08. The horizontal black line indicates the nominal significance threshold (*p* = 0.05), whereas the horizontal grey line indicates the Bonferroni-corrected threshold applied to post hoc comparisons (*p* = 0.0166). Grey bars at the bottom indicate time intervals remaining significant after Holm correction across time points within the analyzed post hoc time window. *p*-values displayed as 0 correspond to values below the numerical resolution afforded by the 10,000 permutations and should therefore be interpreted as *p* < 0.0001; Figure S10: Permutation-based post hoc *p*-values across time for the comparison between the Pre and During2 conditions at electrode CP3 in the Music group. Both panels display the same analyzed time window but with complementary *p*-value ranges. The upper panel focuses on *p*-values above 0.08, whereas the lower panel provides a magnified view of the lower *p*-value range from 0 to 0.08. The horizontal black line indicates the nominal significance threshold (*p* = 0.05), whereas the horizontal grey line indicates the Bonferroni-corrected threshold applied to post hoc comparisons (*p* = 0.0166). Grey bars at the bottom indicate time intervals remaining significant after Holm correction across time points within the analyzed post hoc time window. *p*-values displayed as 0 correspond to values below the numerical resolution afforded by the 10,000 permutations and should therefore be interpreted as *p* < 0.0001; Tables S1: Peak amplitudes and corresponding latencies of somatosensory evoked potentials (SEPs) in the Noise group across four conditions: before (Pre), during (During1 and During2), and after (Post) pink noise exposure. Values are presented as mean ± standard deviation (SD); Table S2: Peak amplitudes and corresponding latencies of somatosensory evoked potentials (SEPs) in the Music group across four conditions: before (Pre), during (During1 and During2), and after (Post) music exposure. Values are presented as mean ± standard deviation (SD); Table S3: Peak amplitudes and corresponding latencies of somatosensory evoked potentials (SEPs) in the Control group across three consecutive recordings (1st, 2nd, and 3rd) temporally aligned with the sequence of the Music and Noise groups (Pre, During1, and During2). Values are presented as mean ± standard deviation (SD).
