# Supplementary material for: Modulation of Somatosensory Evoked Potentials During Music and Pink Noise Exposure
Source: Brain Sci. 2026 Jul 30;16(8):802. doi: 10.3390/brainsci16080802 (PMC13510778; doi:10.3390/brainsci16080802)
Supplement: Supplementary file 1 [file brainsci-16-00802-s001.zip › Figures S1 to S6 Permutation-based ANOVA p-values.pdf]

Figure S1. Permutation-based ANOVA p-values across time for the SEP analysis at electrode F1 in the Noise group. Both panels display the same analyzed time window, but with complementary p-value ranges. The upper panel focuses on p-values above 0.08, whereas the lower panel provides a magnified view of the lower p-value range (0 - 0.08). The horizontal black line indicates the significance threshold (p = 0.05). Grey bars at the bottom indicate time intervals showing a significant main effect of condition after Holm correction for multiple comparisons across time; (p-values displayed as 0 correspond to values below the numerical resolution afforded by the 10,000 permutations and should therefore be interpreted as p < 0.0001).

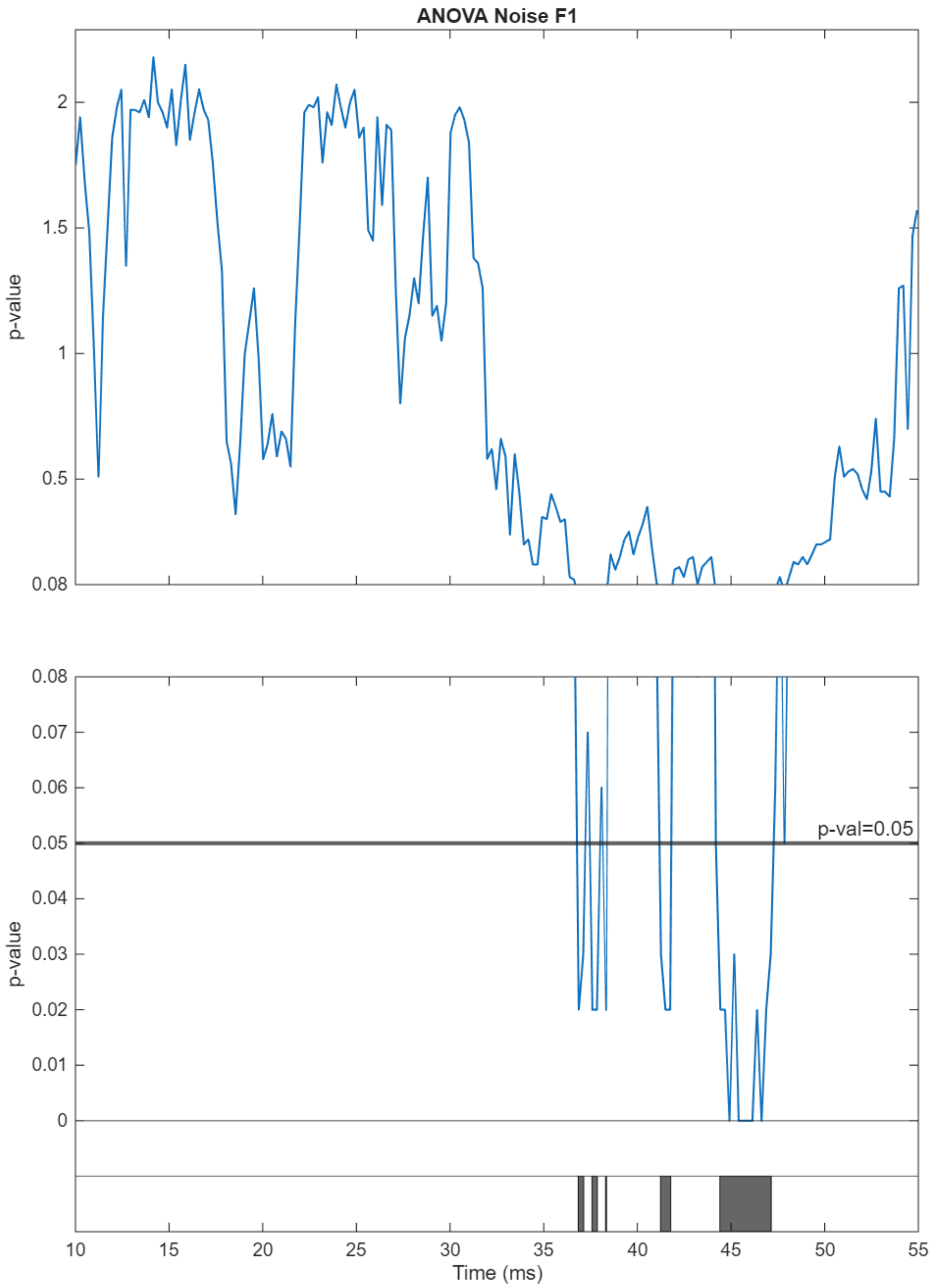

Figure S1

Figure S2. Permutation-based ANOVA p-values across time for the SEP analysis at electrode C1 in the Noise group. Both panels display the same analyzed time window, but with complementary p-value ranges. The upper panel focuses on p-values above 0.08, whereas the lower panel provides a magnified view of the lower p-value range (0 - 0.08). The horizontal black line indicates the significance threshold ($p = 0.05$). Grey bars at the bottom indicate time intervals showing a significant main effect of condition after Holm correction for multiple comparisons across time; (p-values displayed as 0 correspond to values below the numerical resolution afforded by the 10,000 permutations and should therefore be interpreted as $p < 0.0001$).

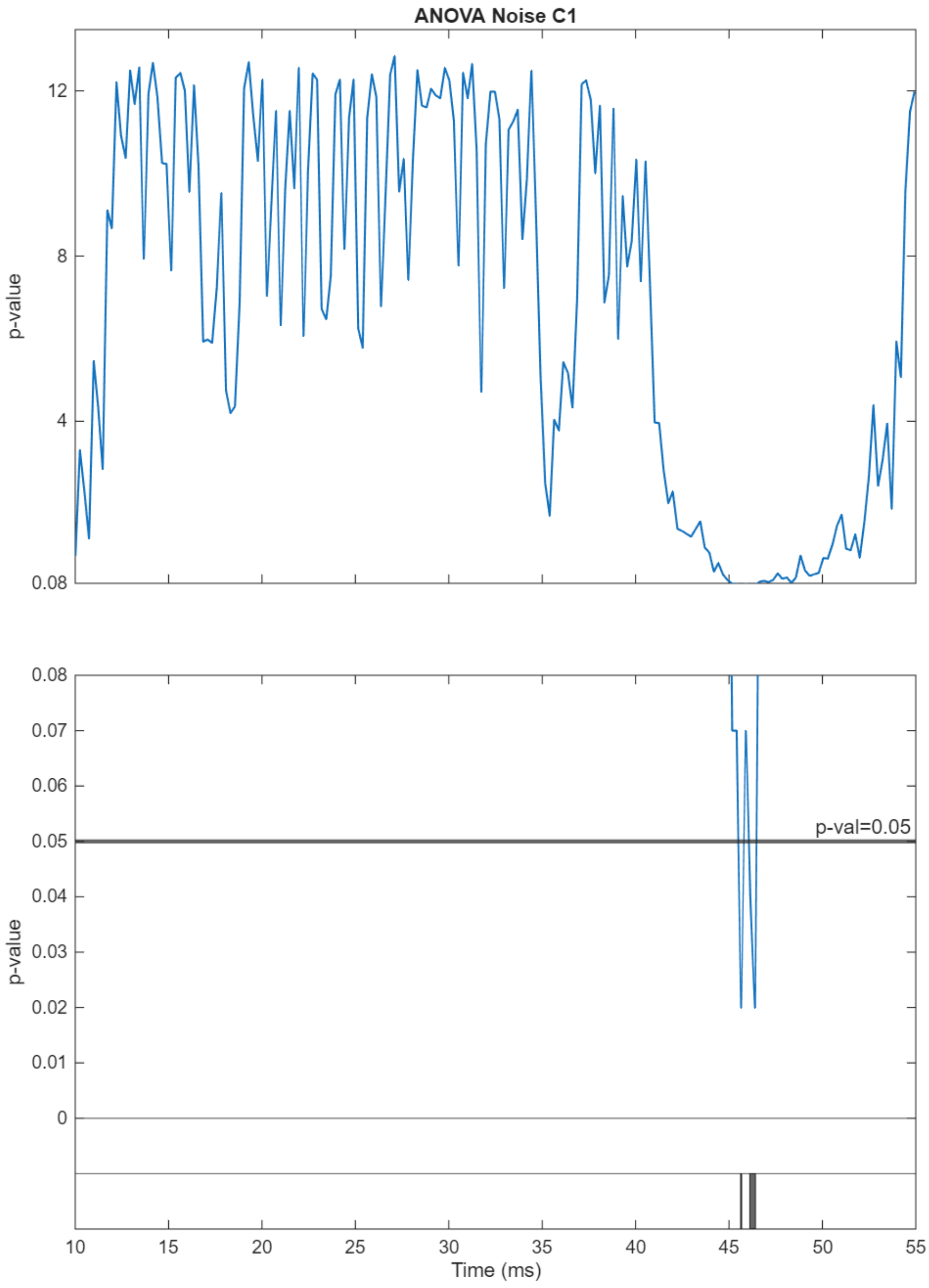

Figure S2

Figure S3. Permutation-based ANOVA p-values across time for the SEP analysis at electrode CP3 in the Music group. Both panels display the same analyzed time window, but with complementary p-value ranges. The upper panel focuses on p-values above 0.08, whereas the lower panel provides a magnified view of the lower p-value range (0 - 0.08). The horizontal black line indicates the significance threshold (p = 0.05). Grey bars at the bottom indicate time intervals showing a significant main effect of condition after Holm correction for multiple comparisons across time; (p-values displayed as 0 correspond to values below the numerical resolution afforded by the 10,000 permutations and should therefore be interpreted as p < 0.0001).

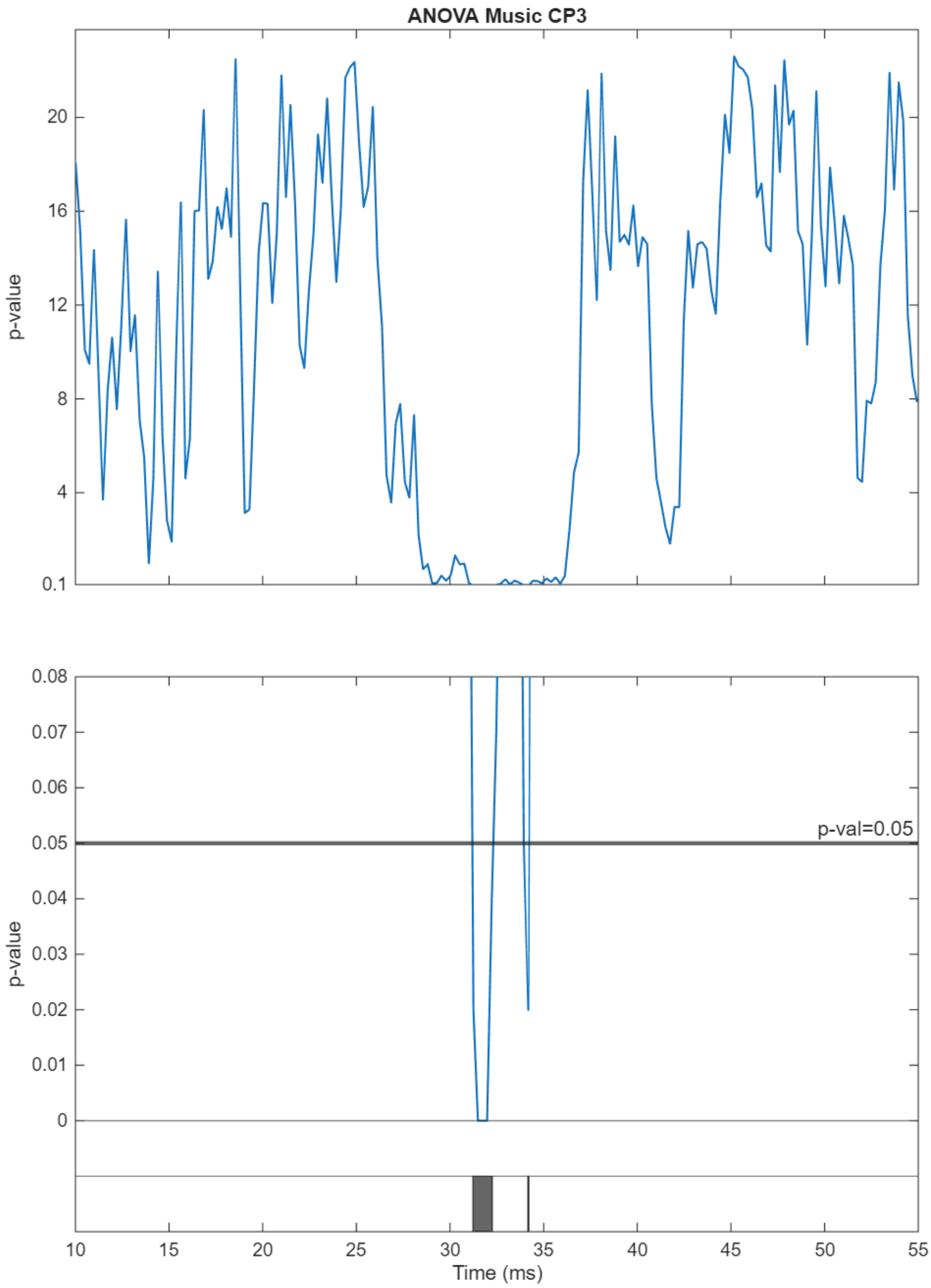

Figure S3

Figures S4, S5 and S6. Permutation-based ANOVA p-values across time for the SEP analysis at electrodes F1, C1, and CP3, respectively, in the Control group. Both panels display the same analyzed time window but with complementary p-value ranges. The upper panel focuses on p-values above 0.08, whereas the lower panel provides a magnified view of the lower p-value range (0–0.08). The horizontal black line indicates the significance threshold ($p = 0.05$). No time intervals survived Holm correction for multiple comparisons across time. P-values displayed as 0 correspond to values below the numerical resolution afforded by the 10,000 permutations and should therefore be interpreted as $p < 0.0001$.

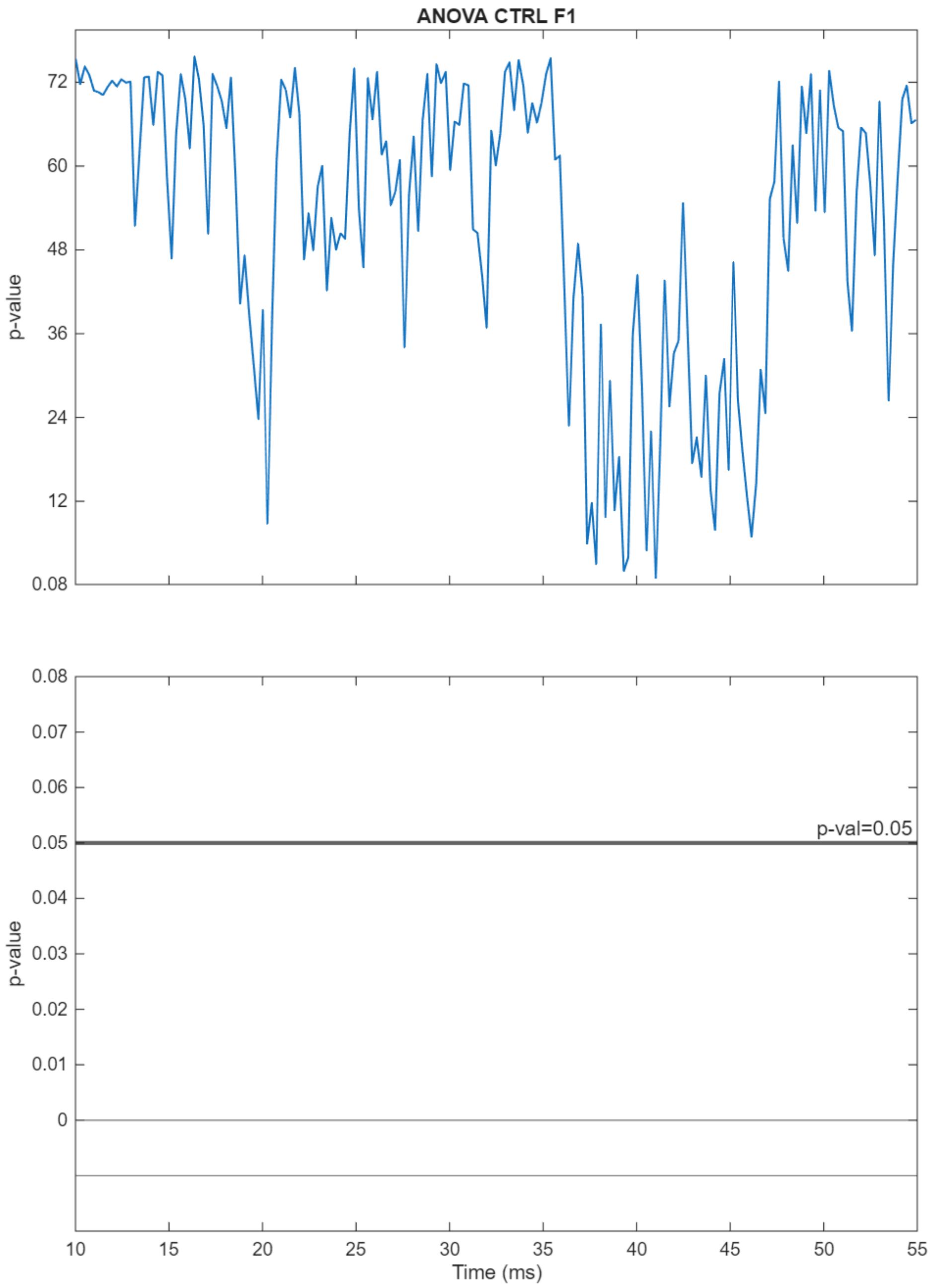

Figure S4

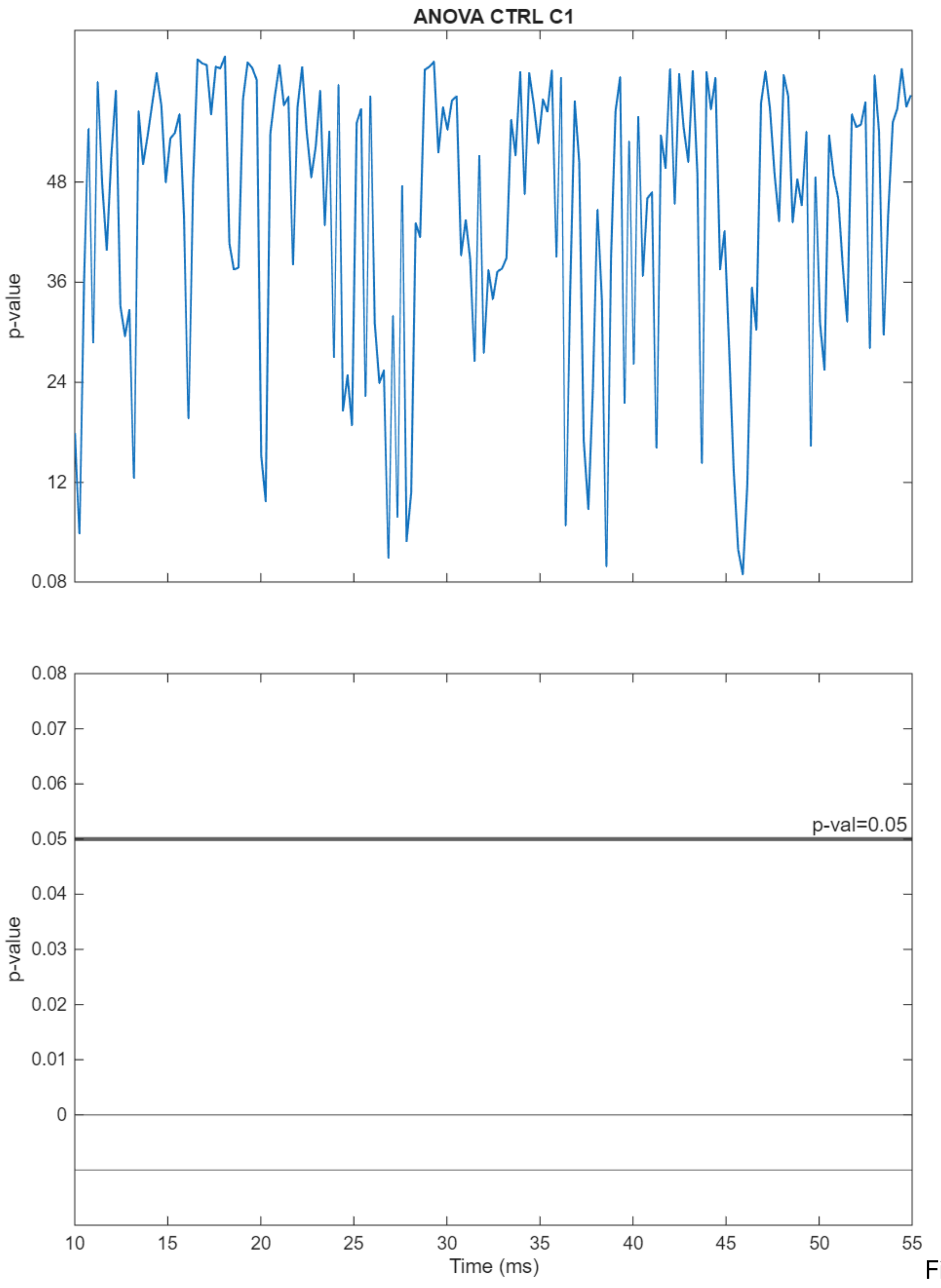
ANOVA CTRL C1
p-value
48
36
24
12
0.08
0.07
0.06
0.05
0.04
0.03
0.02
0.01
0
p-val=0.05
10
15
20
25
30
35
40
45
50
55
Time (ms)

Figure S5

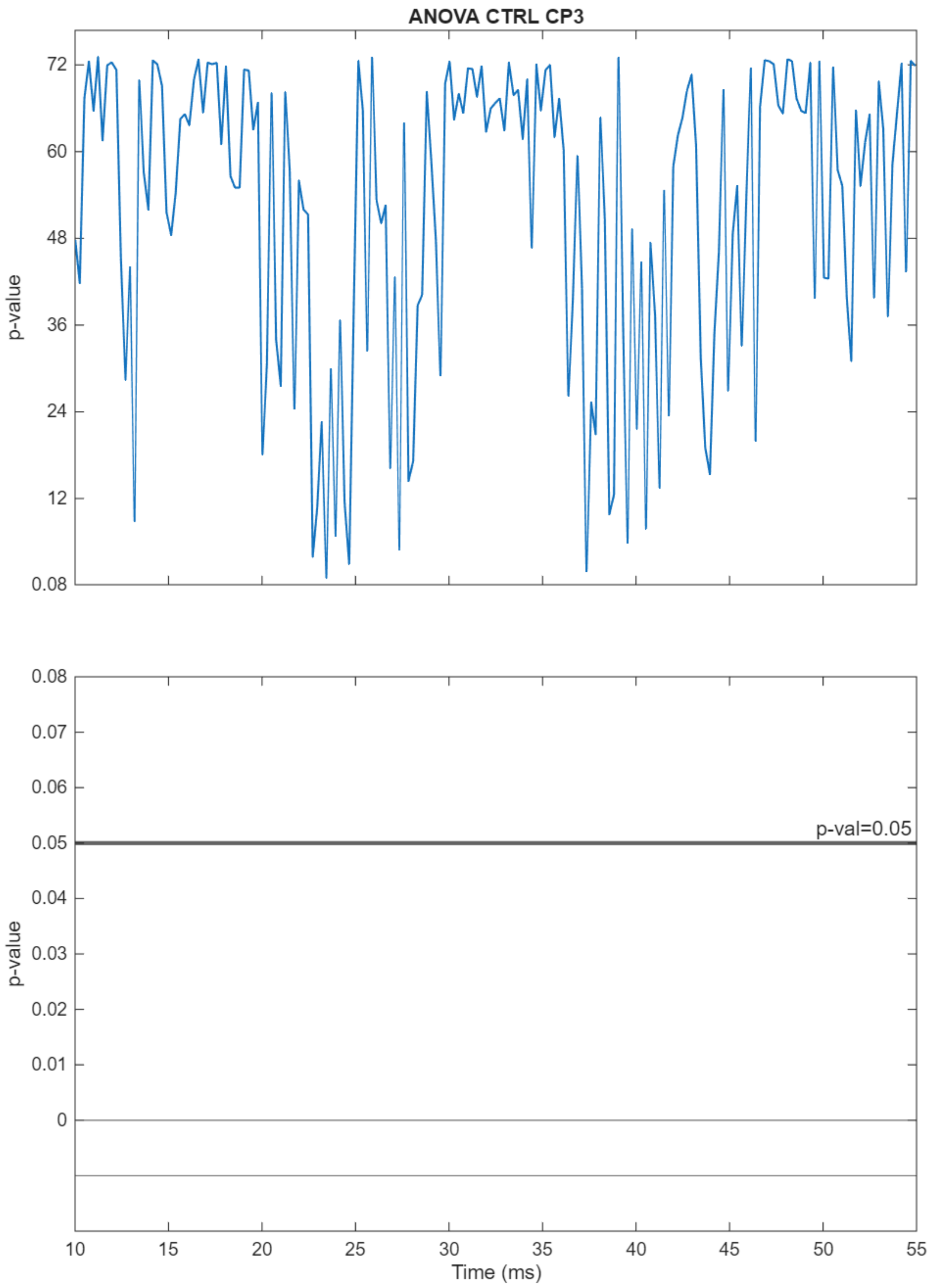
ANOVA CTRL CP3
72
60
48
36
24
12
0.08
p-value
0.08
0.07
0.06
0.05
0.04
0.03
0.02
0.01
0
p-value
p-val=0.05
10
15
20
25
30
35
40
45
50
55
Time (ms)

Figure S6
