# Supplementary material for: Modulation of Somatosensory Evoked Potentials During Music and Pink Noise Exposure
Source: Brain Sci. 2026 Jul 30;16(8):802. doi: 10.3390/brainsci16080802 (PMC13510778; doi:10.3390/brainsci16080802)
Supplement: Supplementary file 1 [file brainsci-16-00802-s001.zip › Figures S7 to S10 Permutation-based post hoc p-values.pdf]

Figure S7. Permutation-based post hoc p-values across time for the comparison between the Pre and During1 conditions at electrode F1 in the Noise group. Both panels display the same analyzed time window but with complementary p-value ranges. The upper panel focuses on p-values above 0.08, whereas the lower panel provides a magnified view of the lower p-value range from 0 to 0.08. The horizontal black line indicates the nominal significance threshold (p = 0.05), whereas the horizontal grey line indicates the Bonferroni-corrected threshold applied to planned post hoc comparisons (p = 0.0166). Grey bars at the bottom indicate time intervals remaining significant after Holm correction across time points within the analyzed post hoc time window. P-values displayed as 0 correspond to values below the numerical resolution afforded by the 10,000 permutations and should therefore be interpreted as p < 0.0001.

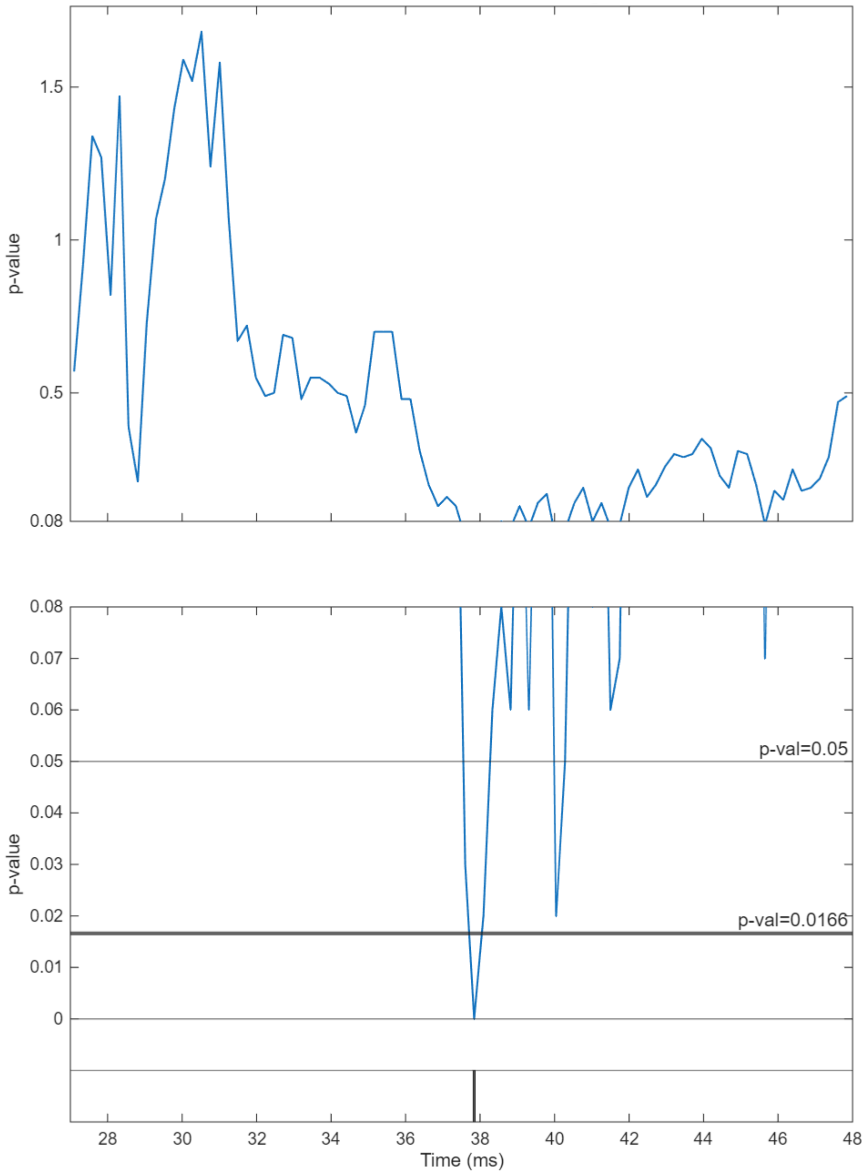

Figure S7

Figure S8. Permutation-based post hoc p-values across time for the comparison between the Pre and During2 conditions at electrode F1 in the Noise group. Both panels display the same analyzed time window but with complementary p-value ranges. The upper panel focuses on p-values above 0.08, whereas the lower panel provides a magnified view of the lower p-value range from 0 to 0.08. The horizontal black line indicates the nominal significance threshold ($p = 0.05$), whereas the horizontal grey line indicates the Bonferroni-corrected threshold applied to post hoc comparisons ($p = 0.0166$). Grey bars at the bottom indicate time intervals remaining significant after Holm correction across time points within the analyzed post hoc time window. P-values displayed as 0 correspond to values below the numerical resolution afforded by the 10,000 permutations and should therefore be interpreted as $p < 0.0001$.

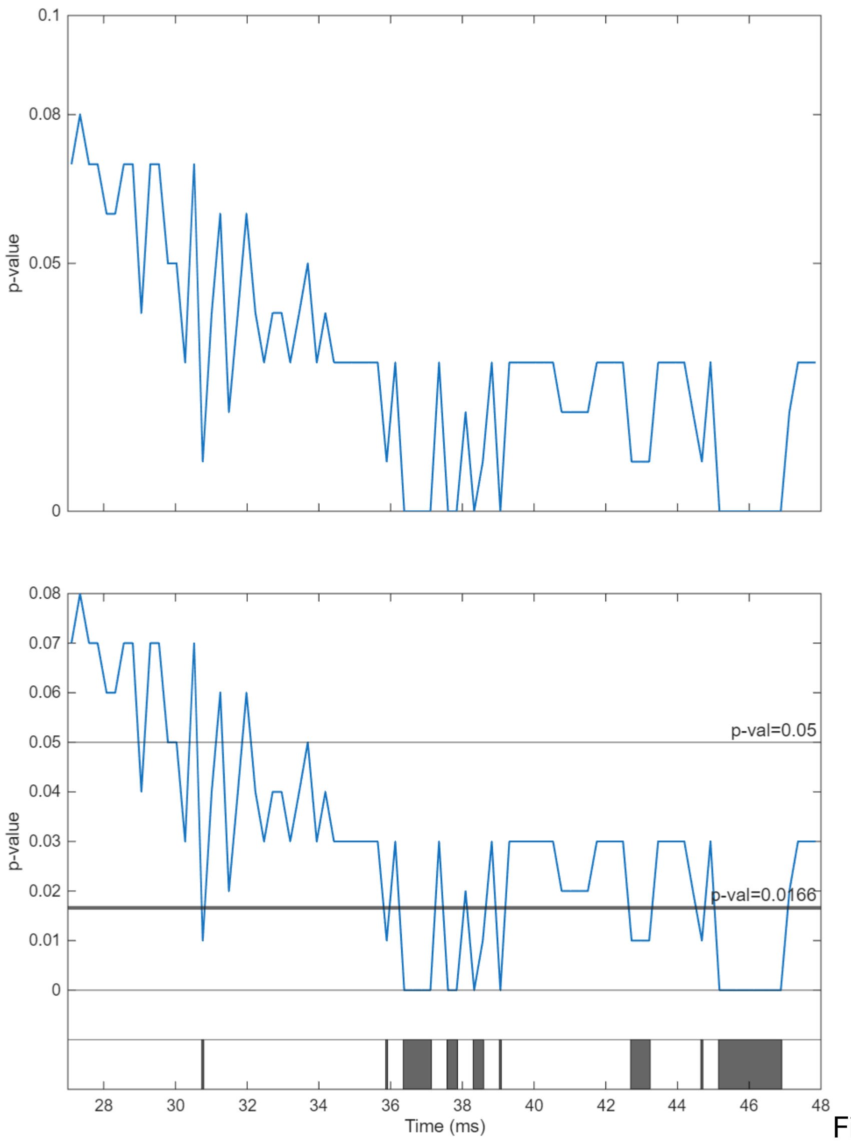

Figure S8

Figure S9. Permutation-based post hoc p-values across time for the comparison between the Pre and During2 conditions at electrode C1 in the Noise group. Both panels display the same analyzed time window but with complementary p-value ranges. The upper panel focuses on p-values above 0.08, whereas the lower panel provides a magnified view of the lower p-value range from 0 to 0.08. The horizontal black line indicates the nominal significance threshold ($p = 0.05$), whereas the horizontal grey line indicates the Bonferroni-corrected threshold applied to post hoc comparisons ($p = 0.0166$). Grey bars at the bottom indicate time intervals remaining significant after Holm correction across time points within the analyzed post hoc time window. P-values displayed as 0 correspond to values below the numerical resolution afforded by the 10,000 permutations and should therefore be interpreted as $p < 0.0001$.

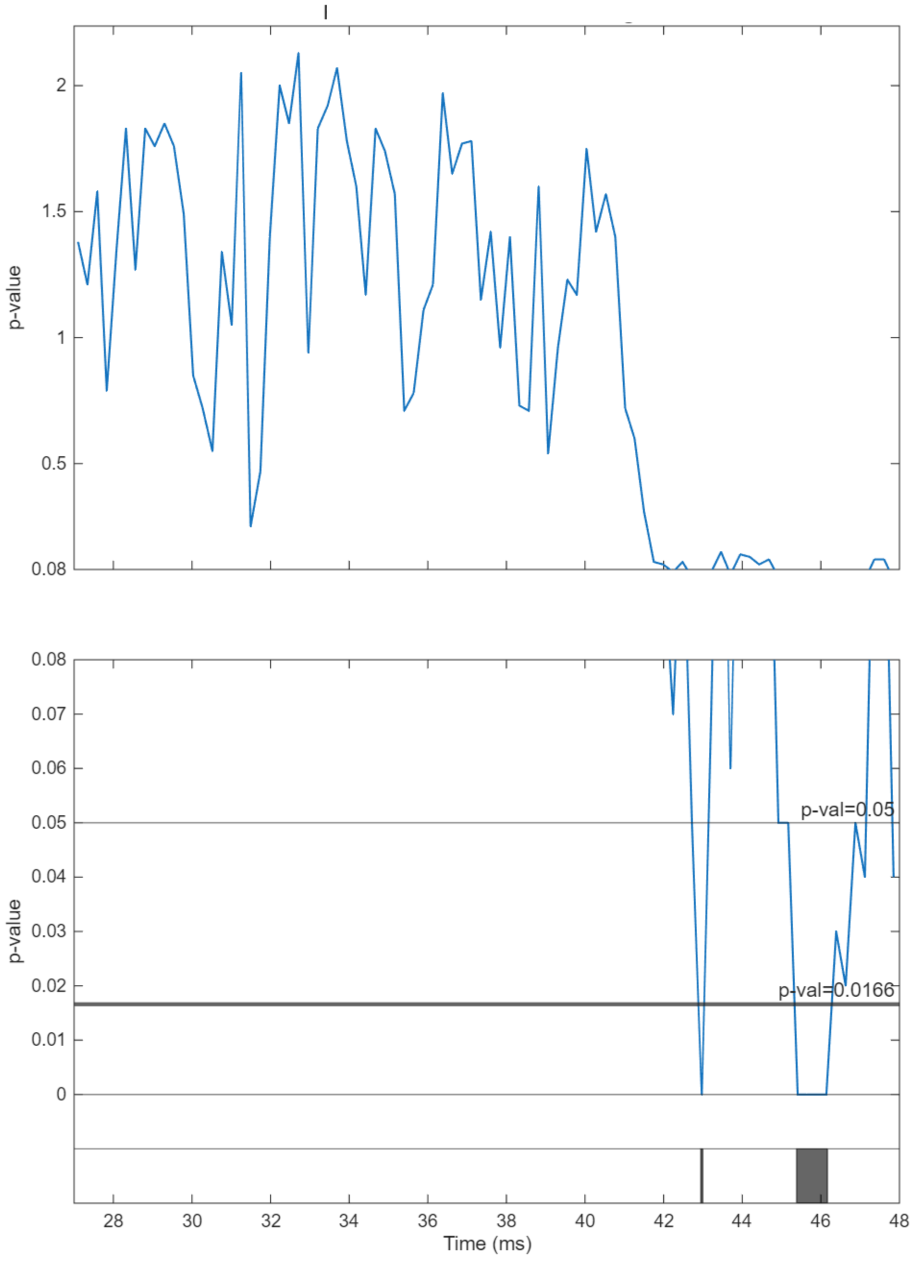

Figure S9

Figure S10. Permutation-based post hoc p-values across time for the comparison between the Pre and During2 conditions at electrode CP3 in the Music group. Both panels display the same analyzed time window but with complementary p-value ranges. The upper panel focuses on p-values above 0.08, whereas the lower panel provides a magnified view of the lower p-value range from 0 to 0.08. The horizontal black line indicates the nominal significance threshold (p = 0.05), whereas the horizontal grey line indicates the Bonferroni-corrected threshold applied to post hoc comparisons (p = 0.0166). Grey bars at the bottom indicate time intervals remaining significant after Holm correction across time points within the analyzed post hoc time window. P-values displayed as 0 correspond to values below the numerical resolution afforded by the 10,000 permutations and should therefore be interpreted as p < 0.0001.

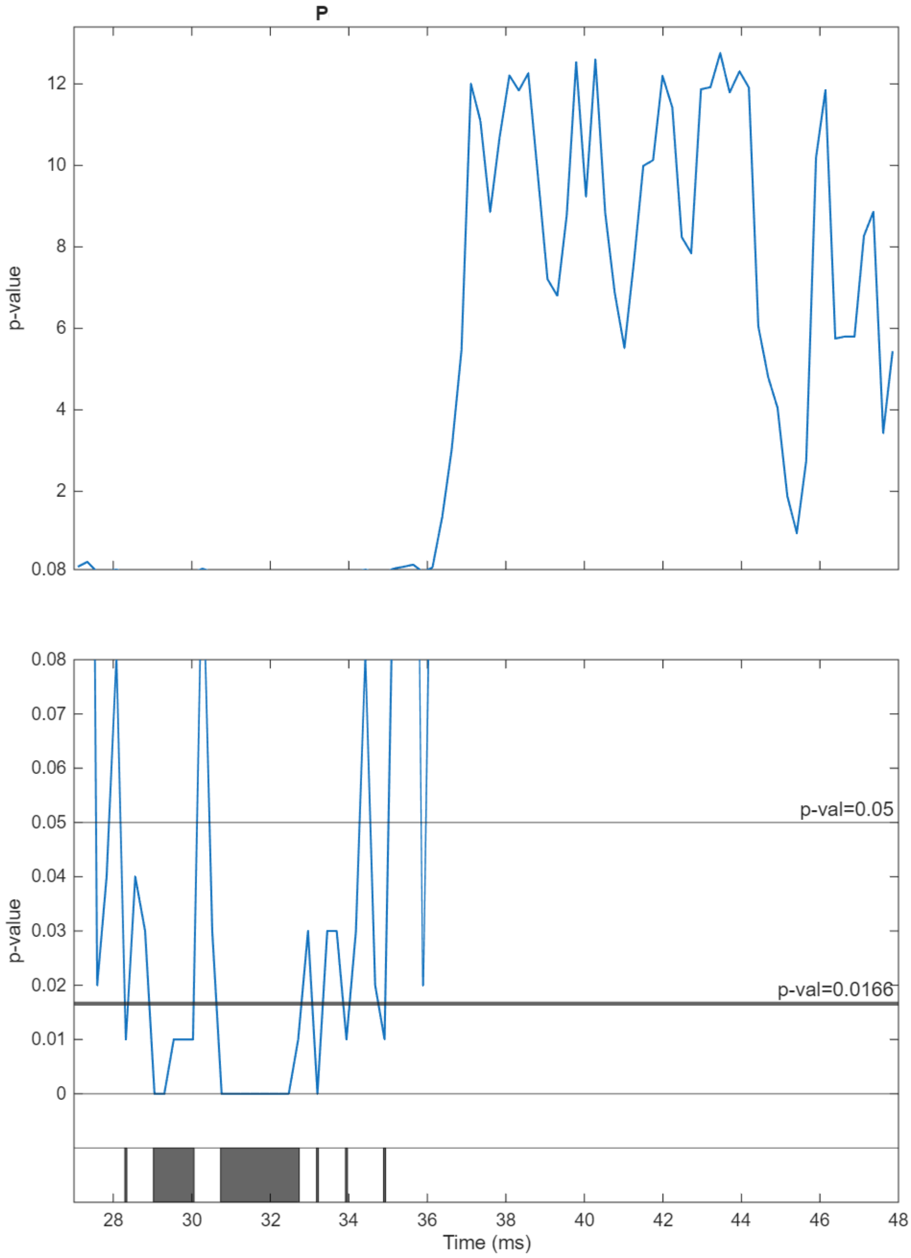

Figure S10
