# Supplementary material for: Modulation of Somatosensory Evoked Potentials During Music and Pink Noise Exposure
Source: Brain Sci. 2026 Jul 30;16(8):802. doi: 10.3390/brainsci16080802 (PMC13510778; doi:10.3390/brainsci16080802)
Supplement: Supplementary file 1 [file brainsci-16-00802-s001.zip › Tables S1-S3.pdf]

Tables S1–S3 provide descriptive information regarding SEP peak amplitudes and latencies. Inferential statistical analyses were performed on the continuous SEP waveforms using permutation-based analyses across predefined temporal windows rather than on isolated peak values.

Table S1. Peak amplitudes and corresponding latencies of somatosensory evoked potentials (SEPs) in the Noise group across four conditions: before (Pre), during (During1 and During2), and after (Post) pink noise exposure. Values are presented as mean ± standard deviation (SD).

Component	Measure	Pre	During1	During2	Post
P14	Amplitude (μV)	1.01 ± 0.40	1.13 ± 0.54	1.17 ± 0.32	1.14 ± 0.52
	Latency (ms)	13.95 ± 0.38	13.95 ± 0.38	13.86 ± 0.37	13.97 ± 0.26
N20	Amplitude (μV)	−0.91 ± 0.64	−0.81 ± 0.64	−0.84 ± 0.56	−1.10 ± 0.70
	Latency (ms)	18.80 ± 0.93	18.93 ± 0.95	19.12 ± 0.90	19.32 ± 0.99
N30	Amplitude (μV)	−2.41 ± 1.47	−2.45 ± 1.30	−2.41 ± 1.56	−2.10 ± 1.41
	Latency (ms)	30.54 ± 1.43	30.24 ± 1.40	30.44 ± 1.21	30.26 ± 1.29
P45	Amplitude (μV)	3.08 ± 1.88	3.57 ± 2.01	3.77 ± 1.96	3.62 ± 1.87
	Latency (ms)	44.02 ± 1.13	44.25 ± 1.34	44.34 ± 1.21	44.74 ± 1.48

Table S2. Peak amplitudes and corresponding latencies of somatosensory evoked potentials (SEPs) in the Music group across four conditions: before (Pre), during (During1 and During2), and after (Post) music exposure. Values are presented as mean ± standard deviation (SD).

Component	Measure	Pre	During1	During2	Post
P14	Amplitude (μV)	1.19 ± 0.39	1.30 ± 0.41	1.19 ± 0.69	1.28 ± 0.45
	Latency (ms)	13.87 ± 0.34	13.82 ± 0.33	13.95 ± 0.41	13.98 ± 0.37
N20	Amplitude (μV)	−1.11 ± 0.75	−1.27 ± 0.81	−1.21 ± 0.82	−1.34 ± 0.94
	Latency (ms)	18.85 ± 0.98	19.21 ± 1.06	19.03 ± 0.92	19.21 ± 1.01
N30	Amplitude (μV)	−2.10 ± 1.41	−2.10 ± 1.61	−2.10 ± 1.44	−2.08 ± 1.55
	Latency (ms)	30.44 ± 1.37	30.03 ± 1.46	30.36 ± 1.38	30.13 ± 1.39
P45	Amplitude (μV)	2.14 ± 1.55	2.65 ± 1.45	2.97 ± 1.30	3.08 ± 1.67
	Latency (ms)	43.68 ± 1.00	44.43 ± 1.57	44.34 ± 1.52	44.22 ± 1.27

Table S3. Peak amplitudes and corresponding latencies of somatosensory evoked potentials (SEPs) in the Control group across three consecutive recordings (1st, 2nd, and 3rd) temporally aligned with the sequence of the Music and Noise groups (Pre, During1, and During2). Values are presented as mean ± standard deviation (SD).

Component	Measure	1st	2nd	3rd
P14	Amplitude (μV)	1.02 ± 0.49	0.95 ± 0.56	0.86 ± 0.57
	Latency (ms)	12.71 ± 0.61	12.70 ± 0.73	12.99 ± 0.54
N20	Amplitude (μV)	−0.92 ± 0.85	−1.19 ± 0.82	−1.00 ± 0.77
	Latency (ms)	19.22 ± 1.23	19.27 ± 1.05	19.22 ± 1.01
N30	Amplitude (μV)	−1.98 ± 1.49	−2.15 ± 1.48	−2.24 ± 1.41
	Latency (ms)	30.32 ± 1.56	29.88 ± 1.46	29.98 ± 1.55
P45	Amplitude (μV)	2.87 ± 1.63	3.10 ± 1.31	3.23 ± 1.29
	Latency (ms)	45.36 ± 1.54	44.78 ± 1.73	44.65 ± 1.46
